# Study on the effects of stand density management of Chinese fir plantation in Northern China

**DOI:** 10.3389/fpls.2023.1130299

**Published:** 2023-05-09

**Authors:** Kun Sun, Honggang Sun, Guobin Lu, Lejen Fang, Zhibing Wan, Zifeng Tan

**Affiliations:** ^1^ Research Institute of Subtropical Forestry, Chinese Academy of Forestry, Hangzhou, China; ^2^ College of Forestry, Beihua University, Jilin, China; ^3^ Yixian Forestry Bureau, Huangshan Forestry Burea, Huangshan, Anhui, China; ^4^ College of Life and Environmental Science, Huangshan University, Huangshan, China

**Keywords:** Chinese fir, thinning, structural heterogeneity, stand diameter distribution, forest productivity

## Abstract

The aim of this study was to clarify the mechanism by which thinning alters stand structure and affects forest productivity by characterizing changes in stand quantitative maturity age, stand diameter distribution, structural heterogeneity, and forest productivity of Chinese fir plantations at different thinning times and intensities. Our findings provide insights into how the density of stands could be modified to enhance the yield and timber quality of Chinese fir plantations. The significance of differences in individual tree volume, stand volume, and timber merchantable volume was determined using one-way analysis of variance and *post hoc* Duncan tests. The stand quantitative maturity age was obtained using the Richards equation. The quantitative relationship between stand structure and productivity was determined using a generalized linear mixed model. We found that (1) the quantitative maturity age of Chinese fir plantations increased with thinning intensity, and the quantitative maturity age was much greater under commercial thinning than under pre-commercial thinning. (2) Individual tree volume and the proportion of medium-sized and large-sized timber merchantable volume increased with stand thinning intensity. Thinning promoted increases in stand diameter. pre-commercially thinned stands were dominated by medium-diameter trees when the quantitative maturity age was reached, whereas commercially thinned stands were dominated by large-diameter trees. The living trees volume will decrease immediately after thinning, and then it will gradually increase with the age of the stand. When the stand volume included both living trees volume and thinned volume, thinned stands increased stand volume compared with unthinned stands. In pre-commercial thinning stands, the greater the intensity of thinning, the greater the increase in stand volume, and the opposite was true for commercial thinning. (3) Thinning also reduced heterogeneity in stand structure, which was lower after commercial thinning than after pre-commercial thinning. The productivity of pre-commercially thinned stands increased with thinning intensity, whereas that of commercially thinned stands decreased with thinning intensity. (4) The structural heterogeneity of pre-commercially and commercially thinned stands was negatively and positively correlated with forest productivity, respectively. In the Chinese fir plantations in the hilly terrain of the northern Chinese fir production area, when pre-commercial thinning was performed in the ninth year to a residual density of 1750 trees per hectare, the stand quantitative maturity age was reached in year 30, medium-sized timber accounted for 75.2% of all trees, and the stand volume was 667.9 m^3^ per hectare. This thinning strategy is favorable for producing medium-sized Chinese fir timber. When commercial thinning was performed in year 23, the optimal residual density was 400 trees per hectare. When the stand quantitative maturity age was reached in year 31, large-sized timber accounted for 76.6% of all trees, and the stand volume was 574.5 m^3^ per hectare. This thinning strategy is favorable for producing large-sized Chinese fir timber.

## 1 Introduction

Chinese fir (*Cunninghamia lanceolata* (Lamb.) Hook.) is an economically important tree species in southern China. The forest area and living tree volume of Chinese fir comprise 63.65% and 68.47% of all forest and living trees in China, respectively (National Forestry and Grassland Administration, 2019). However, there is still much room for improvement in the timber output and quality of existing Chinese fir forests given that plantations are often dominated by a large proportion of young and middle-aged trees, and the living tree volume per unit area is often low. Further improvements in timber output and the quality of existing Chinese fir forests are urgently needed.

Stand yield and timber quality are greatly affected by stand structure. Timely and moderate thinning is an effective measure for regulating stand structure (Sheng, 2001; Pelletier and Pitt, 2008; Lu et al., 2020). High-density stands promote natural pruning and reduce the taper of living trees, and high-quality knot-free timber can be harvested during final felling. However, there is strong competition between adjacent trees in high-density stands because of limitations in habitat resources, and this results in greater stand structural heterogeneity. Most of the trees harvested during final felling operations have small diameters. Previous studies have shown that cutting down a certain proportion of poorly growing trees in the early stages of stand closure reduces stand structural heterogeneity and improves the supply of habitat resources, which promotes the radial growth of residual trees, increases individual tree volume, and optimizes the dimensions of timber (Zhang et al., 2005; Lemay et al., 2018; Rossi et al., 2018). However, pre-commercial thinning only temporarily alleviates the limitation of habitat resources on growth, and it does not fully maximize radial growth potential because of persistent competition from neighboring trees. Commercial thinning at the quantitative maturity age to retain a few dominant trees is a feasible approach for improving stand yield and timber quality. It can also increase radial growth and individual tree volume by reducing stand structural heterogeneity and improving the supply of habitat resources (Soares et al., 2017; Forrester, 2019). At the same time, in the management of timber forests, its maturity can be understood as the state in which the growth and development of the forest stand is most in line with the management objectives (Liu et al., 1989). The forest age at this time is the forest mature age, which is the main basis for determining the main cutting age. Under the premise of continuous management, if the maximum stock volume is taken as the main management goal, the corresponding forest maturity is quantitative maturity, which is marked by the average growth of the volume reaching the maximum value, and the age at this time is the quantitative maturity age (Li, 2011). Discussing the maturity of the quantity, which can realize the time period required for the precise cultivation of medium and large-diameter timber under a certain stand density, and can maximize the productivity of the forest land, thereby providing a scientific and quantitative basis for determining the age of main cutting and improving the level of forest management. It has both theoretical significance and practical value. Although both pre-commercial and commercial thinning promote radial growth and timber quality improvement by altering stand structure, they reduce stand volume and increase the quantitative maturity age to varying degrees depending on the thinning intensity. Due to the close relationship between stand density and stand growth, density regulation will have a positive effect of promoting radial growth due to the expansion of growth space resources, and will also have a negative effect of decreasing stand volume due to the reduction of tree number (Tong et al., 2000). However, some studies have found that after 7 years of stand density control, the average stand diameter and individual tree volume increased significantly compared with those before thinning, which made up for the volume lost due to the reduction of the number of trees (Sun et al., 2011), while density control has a significant impact on forest growth. The negative effect of the stand volume is short-term, and the stand volume eventually follows the law of constant yield during the continuous growth process of the stand (Zhang et al., 2006; Guo et al., 2019). The quality of stand wood is closely related to stand density. Generally speaking, the forest with higher density has higher total timber volume, but the volume of medium and large-diameter timber is relatively small, and the proportion of small-diameter timber is relatively large; while the forest with low density is just the opposite (Duan et al., 2004). Density regulation not only thinned down low-quality trees in the stand, but also creates sufficient nutrient space for the reserved trees to promote their growth, which is more conducive to the cultivation of medium and large diameter trees (Chen and Ding, 2004; Zhang et al., 2006). In addition, the growth of Chinese fir plantations promoted by thinning is caused by the indirect effects of stand environmental factors, in which light plays a dominant role (Xu et al., 2019). While thinning reduces the intensity of competition among trees, it also releases a large amount of growth space, allowing individuals to intercept sufficient light radiation, and at the same time, the growth and development environment of understory vegetation and the decomposition of soil nutrients without the canopy are effectively improved (Zhang et al., 2006; Teste et al., 2012; Cheng et al., 2017), under a series of changes in above-ground and underground parts to promote the growth of Chinese fir plantations. At the same time, thinning promotes tree growth not only in Chinese fir plantations, but also in other tree species. Some studies found that pre-commercial thinning significantly increased the ring width of Jack pine, and increased the number of medium and large diameter timber in the stand (Robert et al., 2008); After moderate thinning, the average diameter and timber volume of the spruce plantation were significantly increased compared with the unthinning stand (Pelletier and Pitt, 2008). When the paper birch pure stand (*Betula papyrifera* Marsh.) was tended and thinned at the stand age of 9 years, the average diameter at breast height, tree height and individual tree volume were significantly higher than those of 1000 and 3000 trees per hectare when the stand density was 400 trees per hectare (Simard et al., 2004); thinning also promoted the radial growth of Canadian black spruce and continued to increase in 10a (Vincent et al., 2009). The intensity of tending thinning is also particularly important. In the study of Korean pine (*Pinus koraiensis*) forests in central Korea, it was found that the average stand diameter at breast height and volume growth rate were positively correlated with light thinning, and negatively correlated with heavy thinning (Juhan et al., 2018). Therefore, it is necessary to determine a reasonable stand density under the current site conditions in order to make full use of the forest land productivity and achieve the purpose of promoting the growth of the stand. At present, most of the studies on stand volume and wood quality of Chinese fir plantation thinning are young and middle-aged forests, and the research sites are mainly concentrated in the middle and southern belts of Chinese fir (Tong et al, 2000; Xu et al., 2014; Guo et al., 2019; Lin et al., 2022), but there are relatively few studies on the effects of stand density management in the northern Chinese fir plantation during the cutting age. Here, we analyzed changes in the relationship between the structure and productivity of Chinese fir plantations after thinning. We also analyzed changes in quantitative maturity age, the diameter distribution, and stand growth (individual tree volume, timber merchantable volume, and stand volume) under different thinning times and intensities, as well as their interrelationships, to determine optimal thinning times and intensities. Our findings will help improve the yield and quality of existing Chinese fir plantations in the northern hilly.

## 2 Materials and methods

### 2.1 Experimental site

**Figure 1:**
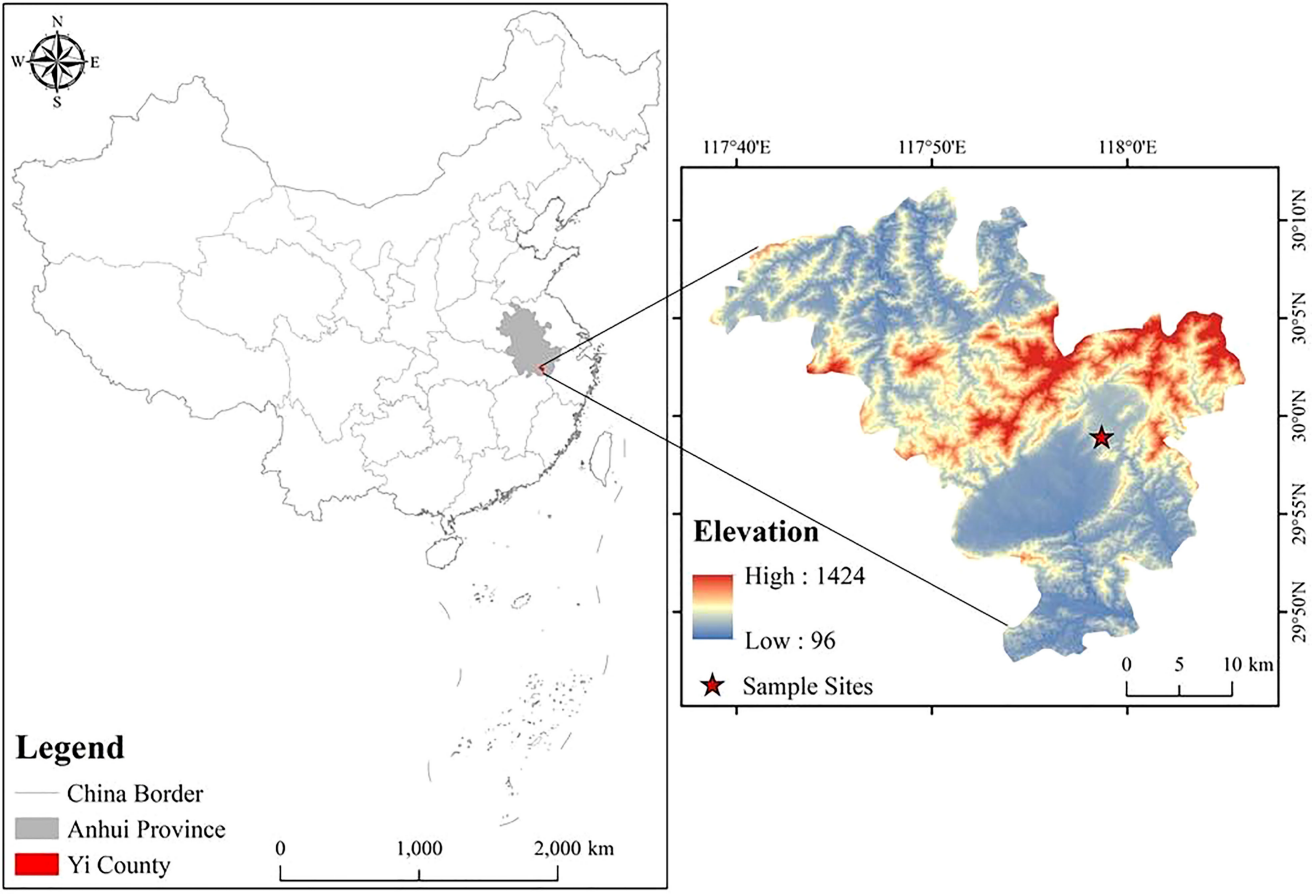
Location map of Hongxing Forest Farm Experimental Area in Yi County, Anhui Province.

Our experiment was conducted at Hongxing Plantation (30°01′N, 117°82′E), Yi County, Anhui Province, China (**Figure 1**). The area experiences a north subtropical humid monsoon climate, with an annual maximum temperature of 27.1°C and a minimum temperature of 3.7°C, an annual average temperature of 15.5°C, an annual effective accumulated temperature of 5113–5226°C, an annual precipitation of 1600–1800 mm, and an annual frost-free period of 235 days. The experimental plots were all located on the lower sunny slope of a hill, with an average slope of 7° and yellow-brown soil. Common understory shrubs include *Litsea cubeba* (Sieb. et Zucc.) Bl, *Loropetalum chinense* (R. Br.) Oliver var. *rubrum* Yieh, *Lespedeza bicolor* Turcz., and *Rhus chinensis* Mill.; common herbs include *Miscanthus floridulus* (Lab.) Warb. ex Schum. et Laut., *Dicranopteris dichotoma* (Thunb.) Berhn., *Odontosoria chinensis* (L.) J. Sm., and *Coniogramme japonica* (Thunb.) Diels; and common vines include *Uncaria rhynchophylla* (Miq.) Miq. ex Havil. and *Pericampylus glaucus* (Lam.) Merr.

### 2.2 Experimental plots

In the winter of 1988, 21 thinning experimental plots of Chinese fir (20.0 m × 20.0 m) were constructed in a Chinese fir clear-felling area; site conditions were the same among all plots. The plantation density of each plot was 5,000 trees per hectare. In the spring of 1989, the survival rate of seedlings was determined, and seedlings from the same batch were used for replanting. In the first three years after they were planted, competing vegetation that affected seedling growth was removed in April and mid-September each year. When the height of the seedlings exceeded 1.30 m, each sapling was numbered and labeled. Diameter at breast height (*D*/cm), tree height (*H*/m), crown width (*CW*/m), crown base height (*CBH*/m), and survival were measured each year.

In the 9th year after plantation, nine plots were randomly selected for pre-commercial thinning to residual stand densities of 3,250 (T_E3250_), 2,500 (T_E2500_), and 1,750 trees per hectare (T_E1750_) (three repetitions for each thinning intensity). No thinning was performed on these plots thereafter. Twenty-three years after seedlings were planted, nine plots were randomly selected from the unthinned plots for commercial thinning, which involved removing all trees except those showing strong growth. The residual stand densities after thinning were 400 (T_L400_), 278 (T_L278_), and 156 trees per hectare (T_L156_) (three repetitions per intensity). The remaining three plots with no thinning were used as controls. Both pre-commercial and commercial thinning were conducted by removing small and poorly growing trees and retaining large, well-growing ones. Both pre-commercial and commercial thinning adopt the method of understory thinning, and both follow the principle of “cutting the small and keeping the big and cutting the bad and keeping the good”.

### 2.3 Measurements

#### 2.3.1 Quantitative maturity age

**Figure 2:**
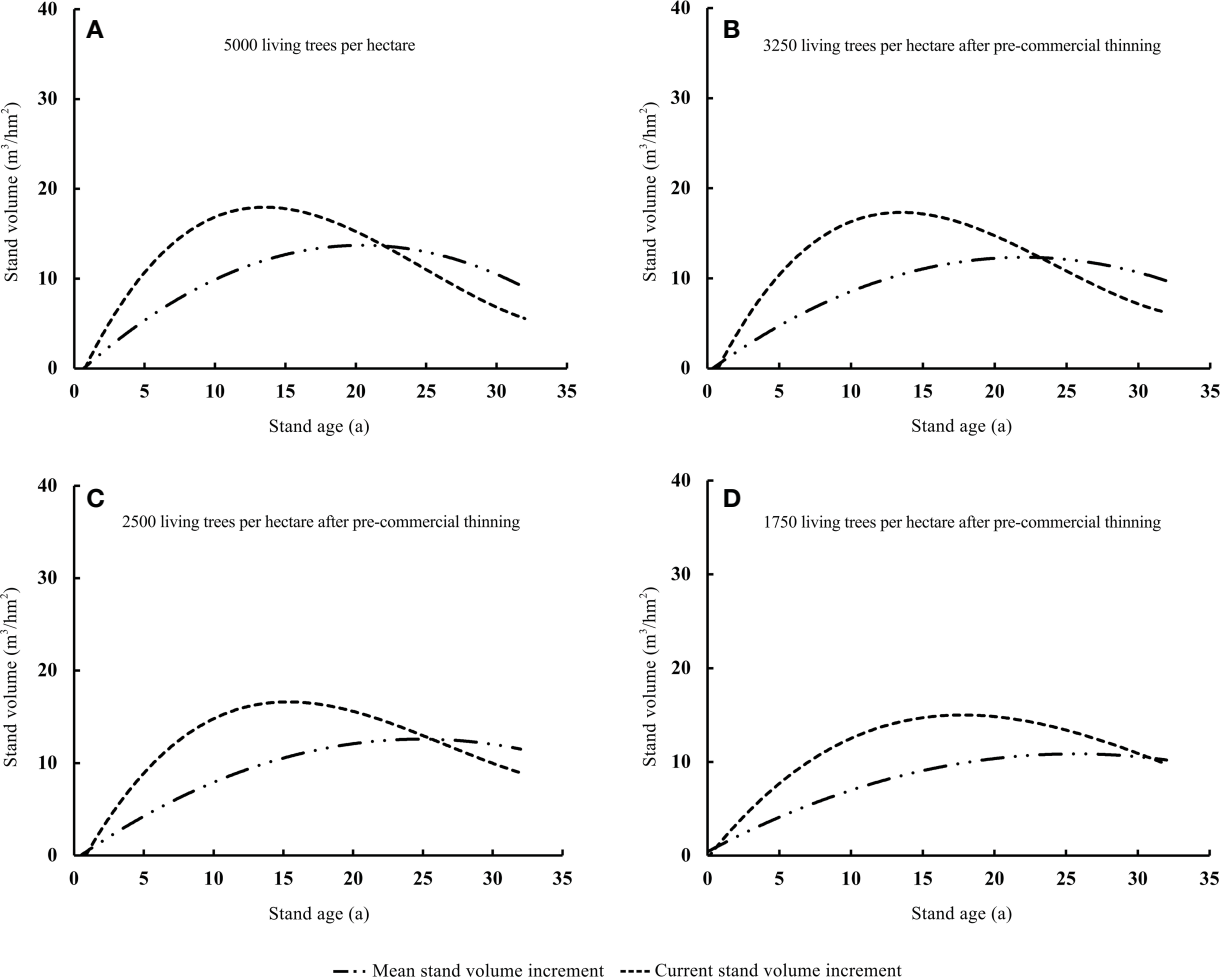
Intersection point (quantitative maturity age) of mean stand volume increment and current stand volume increment under different pre-commercial thinning intensities **(A–D)**.


Use the average standard wood method to select a standard tree in the stand for trunk analysis and use 2m as a section to intercept discs for trunk analysis. The Logistic and Richards theoretical growth models conforming to the “S” type growth curve were selected for fitting, and the data of tree height, diameter at breast height and volume of various ages in the analytical wood were used for inspection, and it was found that the Chinese fir plantation Among them, Richards has the best fitting accuracy (a= 49.508; b= 0.221; c= 2.949; R^2 =^ 0.995, RMSE=0.032), so it is determined to use Richards to simulate the growth process of Chinese fir plantation. Deriving the Richard equation to obtain the annual growth amount and the average growth amount, according to the number of maturity age is the maximum value of the average growth amount of the stand, that is, the average growth of the stand The intersection point when the amount is equal to the annual growth amount is presented by plotting the average growth amount and the annual growth amount (**Figures 1**, **2** in the article).

$$\text{y}=\text{a}{\left(1-{\text{e}}^{\text{rt}}\right)}^{\text{c}}$$

#### 2.3.2 Distribution of the number of trees per diameter class and timber merchantable volume

The number and percentage of living trees per diameter class were calculated using 2 cm as the classification interval. The timber merchantable volume (*V_D_*) of small-sized (8< *D* ≤ 14 cm), medium-sized (16< *D* ≤ 20 cm), and large-sized timber (*D* ≥ 22 cm) was calculated using the following formula (Liu et al., 1995; Liu and Tong, 1980):

*V_D_* = 3.60243758 × 10^-5^ × DBH^1.94752076^ × TH^1.00793769^ (4)

#### 2.3.3 Stand diameter distribution

A normal distribution was used to fit changes in the stand diameter structure. The skewness (*SK*) and kurtosis (*ST*) of the stand diameter distribution were calculated using equations 1 and 2, respectively:


(1) $$SK=\frac{n}{\left(n-1\right)\left(n-2\right)}\sum_{i=1}^{n}{\left[\frac{x_{i}-\bar{x}}{s}\right]}^{3}$$

(2) $$ST=\left\{\frac{n\left(n+1\right)}{\left(n-1\right)\left(n-2\right)\left(n-3\right)}\sum_{i=1}^{n}{\left[\frac{x_{i}-\bar{x}}{s}\right]}^{4}\right\}-\frac{3{\left(n-1\right)}^{2}}{\left(n-2\right)\left(n-3\right)}$$

where n is the number of living trees in the stand, $x_{i}$ is the diameter of living trees in the stand, $\bar{x}$ is the arithmetic mean diameter of the stand, and s is the standard deviation.

#### 2.3.4 Stand structure

The Gini coefficient (GC) was first used to analyze the degree of difference in the income of a certain group of people. Before this study, we carried out the analysis and comparison calculation of five structural parameters. From the results, we found that the GC was found to more accurately measure size inequality in the individual frequency distribution of plant populations, and the GC had advantages in terms of its discriminant power, ability to provide a logical ordering of different distributions, and sensitivity to changes in sample size (Nils and Tron, 2006). The scale invariance of GC allowed it to accurately determine the difference in size inequality among stands, despite the number of trees within stands varying substantially pre-commercial and commercial thinning. The Gini coefficient (*GC*) measures the dispersion of a frequency distribution of individual tree basal area within the stand. The closer *GC* is to 0, the smaller the size difference between living trees in the stand and the lower the structural heterogeneity; the closer *GC* is to 1, the greater the size difference between living trees in the stand and the higher the structural heterogeneity (Zeileis, 2014):


(3) $$GC=\frac{\sum_{j=1}^{n}\left(2j-n-1\right)ba_{j}}{\sum_{j=1}^{n}ba_{j}\left(n-1\right)}$$

where *j* is the rank in ascending order of the cross-sectional area of individual living trees in the stand and $ba_{j}$ is the cross-sectional area of the *j*th living tree.

#### 2.3.5 Forest productivity

Forest productivity was determined through measurements of the periodic annual increase (*PAI*/m^3.^ha^−1^ year^−1^) in stand volume. The total volume of all living trees in the plot was defined as the stand volume (existing volume + thinned volume) (*SV*/m^3^/hm^2^). The empirical formula for calculating the timber volume of Chinese fir living trees (*V*/m^3^) (Liu et al., 1980) was as follows:

*V* = 0.00005877042 × DBH^1.9699831^ × TH^0.89646157^ (5)

#### 2.3.6 Statistical analysis

Differences in measurements under different thinning times and intensities were determined by one-way analysis of variance. Multiple comparisons were performed using Duncan’s tests. The quantitative relationship between stand structure and productivity was analyzed using a generalized linear mixed model. Statistical analyses were conducted, and graphs were built using R 4.2.0 software (Core Team, 2015).

## 3 Results and analysis

### 3.1 Effect of thinning on the quantitative maturity age

**Figure 3:**
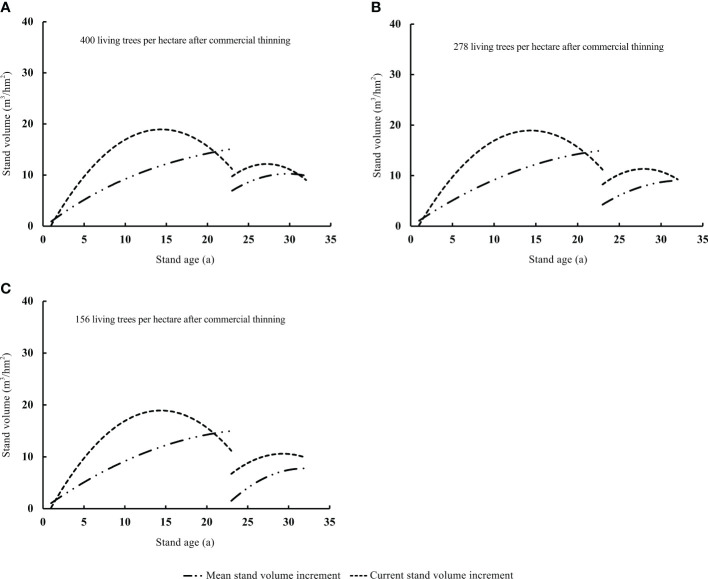
Intersection point (quantitative maturity age) of mean stand volume increment and current stand volume increment under different commercial thinning intensities **(A–D)**.

After thinning, the stand volume immediately decreased and then gradually increased. This process altered the quantitative maturity age of the Chinese fir plantation. The quantitative maturity age was higher under pre-commercial and commercial thinning than in unthinned stands. The increase in quantitative maturity age caused by thinning was positively related to the thinning intensity (**Figures 2**, **3**). In our study, the quantitative maturity age of the unthinned stand was 22 years (**Figure 2A**). The quantitative maturity ages of the stands with residual densities of T_E3250_, T_E2500_, and T_E1750_ after pre-commercial thinning were 23, 26, and 30 years, respectively (**Figures 2B–D**). By contrast, the quantitative maturity age of stands with residual densities of T_L400_ and T_L278_ after commercial thinning were 31 and 32 years, respectively; and the quantitative maturity age of the stand with a residual density of T_L156_ after thinning had not yet been reached during the study period (**Figures 3A–C**). The increase in quantitative maturity age caused by commercial thinning was much longer than that caused by pre-commercial thinning. This also means that the stand age at final felling was much greater with commercial thinning than with pre-commercial thinning.

### 3.2 Effect of thinning on individual tree growth

**Figure 4:**
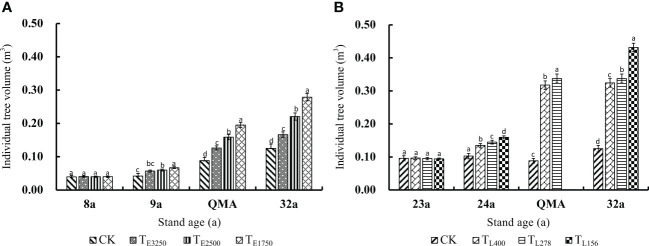
Variation law of individual tree volume in the pre-commercial **(A)** and commercial **(B)** stands with different thinning intensities before thinning, after thinning, quantitative maturity age and the last observation Note: In the **Figures 8A**, **9A**, QMA and 32a are before pre-commercial thinning, after thinning, quantitative maturity age and the last observation, respectively, and T_E3250_, T_E2500_ and T_E1750_ are pre-commercial thinning stand retention densities of 3250, 2500 and 1750 trees per hectare; 23a, 24a, QMA and 32a are before commercial thinning, after thinning, quantitative maturity age and the last observation, respectively, and T_L400_, T_L278_ and T_L156_ are commercial thinning stand retention densities of 400, 278 and 156 trees per hectare. CK is an unthinned stand as a control (Stand density is 5000 trees per hectare). Different lowercase letters indicate significant differences in individual tree volume between residual stand densities at the same stand age (*p*< 0.05). The same applies to the following figures.

Individual tree volume increased with stand age. Thinning further promoted the growth of the individual volume of residual trees (**Figure 4**). Thinning intensity (both *via* pre-commercial thinning and commercial thinning) was positively related to increases in the individual volume of residual trees. However, the increase in individual volume after commercial thinning was much greater than that after pre-commercial thinning. The increase in individual volume at the quantitative maturity age was 86.4% higher after pre-commercial thinning and 314.8% higher after commercial thinning (*p*<0.01). Commercial thinning promoted the growth of individual tree volume more than pre-commercial thinning.

### 3.3 Effects of thinning on diameter class

**Figure 5:**
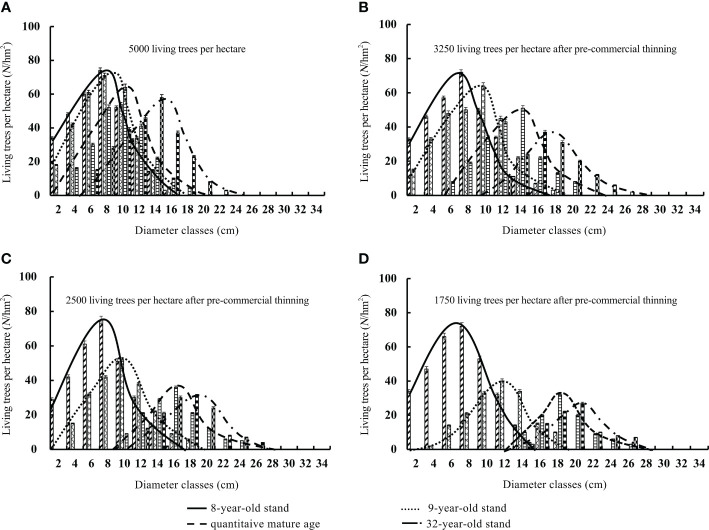
Diameter class distribution of the number of living trees in the stands **(A–D)** with different pre-commercial thinning intensities.

**Figure 6:**
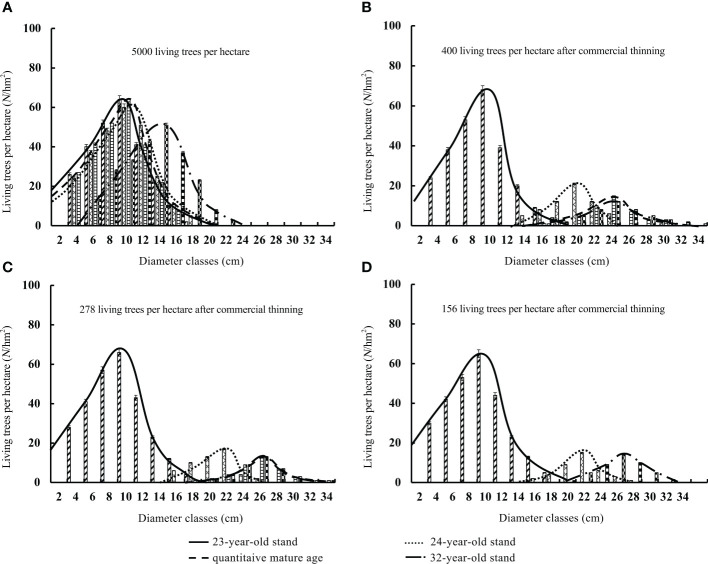
Diameter class distribution of the number of living trees in the stands **(A–D)** with different commercial thinning intensities.

Thinning accelerated increases in stand diameter and altered the stand diameter distribution (**Table 1**). The average diameter of the unthinned stand increased with stand age. The peak of the stand diameter distribution and initial diameter class gradually increased. However, the stand diameter distribution was always left-skewed. When the stand reached the quantitative maturity age, the proportion of small-diameter trees (74.6%) was much larger than that of medium-diameter ones (6.3%) (**Figure 5A**). After pre-commercial thinning, both the skewness and kurtosis of the stand diameter class decreased. The peak of the diameter distribution and the size of the initial diameter class increased rapidly with thinning intensity. The diameter distribution became increasingly right-skewed as the thinning intensity increased. When the stand reached the quantitative maturity age, the proportion of small-diameter living trees gradually decreased from 70.1% to 42.1%; the proportion of medium-diameter trees increased rapidly from 8.5% to 47.8%; and the proportion of large-diameter trees gradually increased from 0% to 8.6% (**Figures 5B–D**). Commercial thinning drastically altered the stand diameter distribution. It changed from left-skewed to right-skewed immediately after thinning, and the kurtosis also decreased sharply. The peak of the diameter distribution and the size of the initial diameter class increased abruptly after thinning. When the stand reached the quantitative maturity age, the proportion of small-diameter living trees sharply decreased from 67.9% to 0%, the proportion of medium-diameter trees increased from 7.8% to 13.2%, and the proportion of large-diameter trees gradually increased from 0% to 90.2% (**Figures 5B–D**). Before thinning, small-diameter trees were most common, followed by medium-diameter trees; after thinning, large-diameter trees were most common, followed by medium-diameter trees (**Figure 6**).

**Table 1:** Effect of thinning on the stand diameter distribution of Chinese fir plantations.

Thinning intensity	Skewness			Kurtosis		
	Before thinning	After thinning	QMA	Before thinning	After thinning	QMA
Control	0.57	0.56	0.31	1.35	1.36	1.13
T_E3250_	0.49	0.39	0.20	1.28	1.03	0.74
T_E2500_	0.39	0.25	0.14	1.15	0.82	0.59
T_E1750_	0.25	-0.11	-0.01	1.01	0.75	0.38
T_L400_	0.29	-0.16	-0.10	1.12	0.46	0.33
T_L278_	0.33	-0.24	-0.15	1.11	0.35	0.23
T_L156_	0.31	-0.27	—	1.06	0.21	—

**Figure 7:**
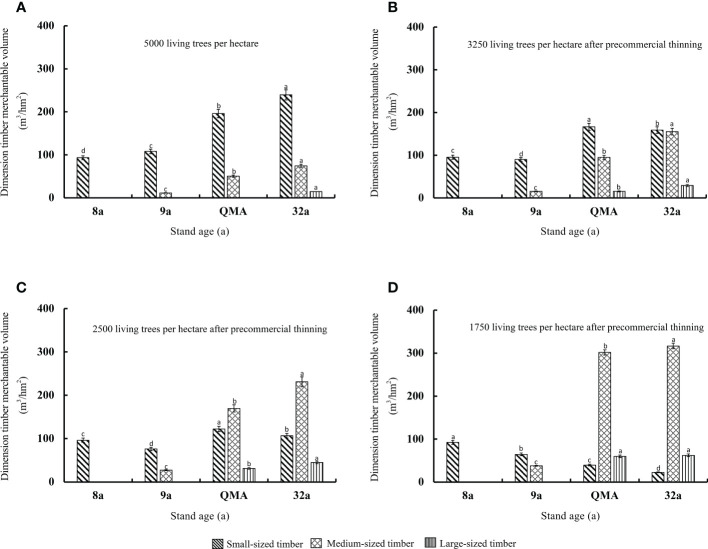
Variations of the timber merchantable volume in stands with different pre-commercial thinning intensities **(A–D)** before and after thinning, at the quantitative maturity age and last observation.

**Figure 8:**
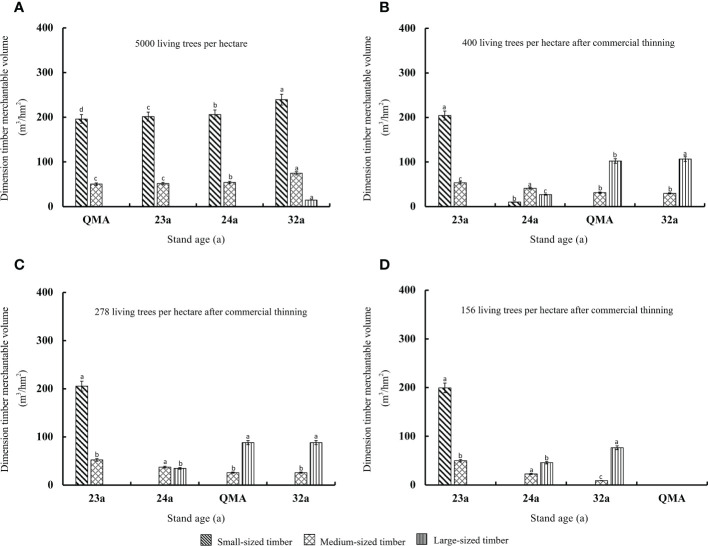
Variations of the timber merchantable volume in stands with different commercial thinning intensities **(A–D)** before and after thinning, at the quantitative maturity age and last observation.

Stand timber merchantable volume (small-sized timber, intermediate-sized timber and large-sized timber) increases continuously with stand age, and thinning will change the types and proportions of standard timber species (**Figures 7**, **8**). In unthinned stands, timber merchantable volume mainly small-sized timber, followed by intermediate-sized timber. When the unthinned stand reached the quantitative maturity age, the small-sized and intermediate-sized timber merchantable volumes were 195.9 and 50.4 m^3^ per hectare, accounting for 79.5% and 19.2% of the timber merchantable volume, respectively. The small-sized timber in pre-commercial and commercial thinning was less than that of the unthinned stand. When the stand reached the quantitative maturity stage, the small-sized timber of T_E3250_, T_E2500_ and T_E1750_ in the pre-commercial thinning was significantly reduced by 15.1%, 37.5% and 79.8% compared with the unthinned stand. The amount was significantly reduced by 100% (*p*<0.05). The higher the intensity of pre-commercial thinning, the higher the intermediate and large-sized timber, but the opposite was true for commercial thinning stands. The intermediate-sized timber in the pre-commercial thinned T_E3250_, T_E2500_ and T_E1750_ was significantly higher than that of the unthinned stand by 87.8%, 236.3% and 498.1%, and the large-sized timber increased significantly by 15.33 m^3^/hm^2^, 31.37 m^3^/hm^2^ and 60.03 m^3^/hm^2^ (*p*<0.05); the large-sized timber in the commercial thinned T_L400_ and T_L278_ was significantly higher than that of the unthinned stand by 102.28 m^3^/hm^2^ and 87.99 m^3^/hm^2^. Pre-commercial thinning stands T_E2500_ and T_E1750_, in which the intermediate-sized timber accounted for 52.4% and 75.2% of the timber merchantable volume; commercial thinning stands T_L400_ and T_L278_, in which the large-sized timber accounted for 76.6% and 77.3% of the timber merchantable volume.

### 3.4 Effect of thinning on stand growth

**Figure 9:**
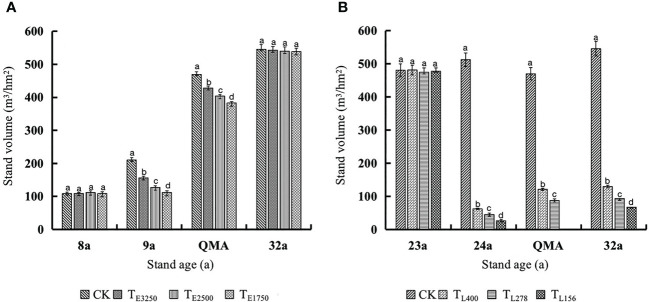
Variation law of stand existing volume in the pre-commercial **(A)** and commercial **(B)** stands with different thinning intensities before thinning, after thinning, quantitative maturity age and the last observation Note: In the **Figures 8A**, **9A**, QMA and 32a are before pre-commercial thinning, after thinning, quantitative maturity age and the last observation, respectively, and T_E3250_, T_E2500_ and T_E1750_ are pre-commercial thinning stand retention densities of 3250, 2500 and 1750 trees per hectare; 23a, 24a, QMA and 32a are before commercial thinning, after thinning, quantitative maturity age and the last observation, respectively, and T_L400_, T_L278_ and T_L156_ are commercial thinning stand retention densities of 400, 278 and 156 trees per hectare. CK is an unthinned stand as a control (Stand density is 5000 trees per hectare).

The tree volume of unthinned stands continuously increased, and unthinned stands had the lowest quantitative maturity age. The stand volume decreased immediately after thinning and then gradually increased with stand age. However, thinning intensity slowed the recovery of stand volume; consequently, thinned stands had a higher quantitative maturity age (**Figure 9**). In our study, increases in stand volume were much greater after commercial thinning than after pre-commercial thinning. However, commercial thinning intensities were much greater than pre-commercial thinning intensities, and late volume increases were smaller than early volume increases; the recovery of stand volume was longer after commercial thinning than after pre-commercial thinning.

**Figure 10:**
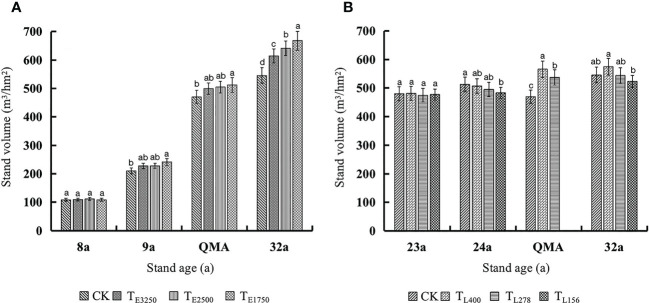
Variation law of stand volume (existing volume + thinned volume) in the pre-commercial **(A)** and commercial **(B)** stands with different thinning intensities before thinning, after thinning, quantitative maturity age and the last observation Note: In the **Figures 8A**, **9A**, QMA and 32a are before pre-commercial thinning, after thinning, quantitative maturity age and the last observation, respectively, and T_E3250_, T_E2500_ and T_E1750_ are pre-commercial thinning stand retention densities of 3250, 2500 and 1750 trees per hectare; 23a, 24a, QMA and 32a are before commercial thinning, after thinning, quantitative maturity age and the last observation, respectively, and T_L400_, T_L278_ and T_L156_ are commercial thinning stand retention densities of 400, 278 and 156 trees per hectare. CK is an unthinned stand as a control (Stand density is 5000 trees per hectare).

When the stand volume includes existing volume + thinned volume, the thinning stand increased the stand volume compared with the unthinned stand (**Figure 10**). In pre-commercial thinning stands, the greater the thinning intensity, the greater the increase in stand volume; in the commercial thinning, the greater the thinning intensity, the smaller the increase in stand volume. Pre-commercial and commercial thinning stand volume was higher than that of unthinned stand at the quantitative maturity age, and pre-commercial thinning stand was significantly higher than that of unthinned stand at 32a. Stand volumes of T_E3250_, T_E2500_ and T_E1750_ were increased by 22.4%, 17.5% and 12.5% respectively; in commercial thinning, only the retention density at T_L400_ increased by 5.3% compared with the unthinned stand (*p*<0.05).

### 3.5 Effects of thinning on stand structure and forest productivity

**Figure 11:**
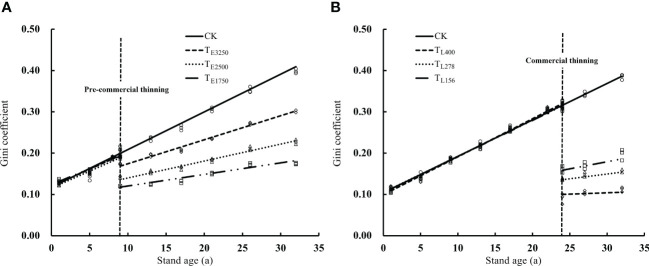
Change trend of stand structure heterogeneity under different pre-commercial **(A)** and commercial **(B)** thinning intensities Note: In the figure, T_E3250_, T_E2500_ and T_E1750_ are pre-commercial thinning stand retention densities of 3250, 2500 and 1750 trees per hectare; T_L400_, T_L278_ and T_L156_ are commercial thinning stand retention densities of 400, 278 and 156 trees per hectare. CK is an unthinned stand as a control (Stand density is 5000 trees per hectare).

The differentiation between living trees in a stand increased with stand age and thinning altered stand structural heterogeneity (**Figure 11**). The structural heterogeneity of unthinned stands increased from the initial 0.10 to 0.39 within a 32-year growth period. Thinning led to a sharp decrease in stand structural heterogeneity, and thinning intensity was inversely related to structural heterogeneity. Compared with unthinned stands, the structural heterogeneity of pre-commercial thinning stands decreased by 30.4%, 37.7%, and 51.5% respectively (*p*<0.05); the Gini coefficients of T_L400_, T_L278_ and T_L156_ decreased significantly by 70.8%, 57.4% and 50.1% (*p*<0.05).Although stand structural heterogeneity increased again with stand age after thinning, it was always smaller than that before thinning and also smaller than that of unthinned stands under the same conditions. In addition, the structural heterogeneity was much smaller after commercial thinning than after pre-commercial thinning. The increase in structural heterogeneity with stand age after commercial thinning was also much smaller than that after pre-commercial thinning. When the stand was 32 years old, the Gini coefficients in T_E3250_, T_E2500_ and T_E1750_ stands were 0.30, 0.23 and 0.19 in pre-commercial thinning, while the Gini coefficients in T_L400_, T_L278_ and T_L156_ stands were 0.20, 0.16 and 0.11 commercial and thinning.

**Figure 12:**
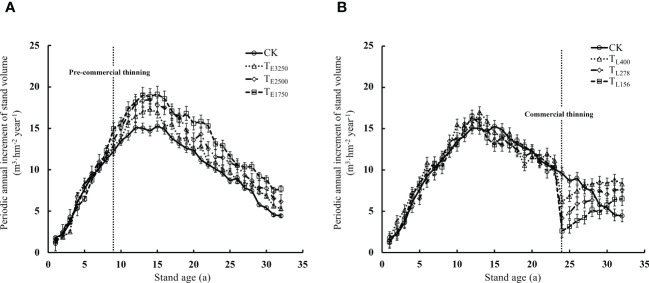
Change trend of forest productivity under different pre-commercial **(A)** and commercial **(B)** thinning intensities Note: In the figure, T_E3250_, T_E2500_ and T_E1750_ are pre-commercial thinning stand retention densities of 3250, 2500 and 1750 trees per hectare; T_L400_, T_L278_ and T_L156_ are commercial thinning stand retention densities of 400, 278 and 156 trees per hectare. CK is an unthinned stand as a control (Stand density is 5000 trees per hectare).

**Figure 13:**
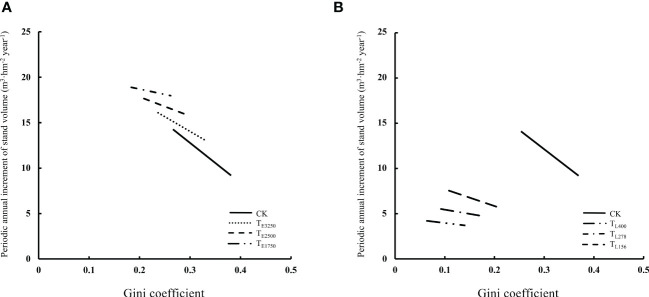
Effect of pre-commercial **(A)** and commercial **(B)** thinning on the relationship between stand structure and forest productivity Note: In the figure, T_E3250_, T_E2500_ and T_E1750_ are pre-commercial thinning stand retention densities of 3250, 2500 and 1750 trees per hectare; T_L400_, T_L278_ and T_L156_ are commercial thinning stand retention densities of 400, 278 and 156 trees per hectare. CK is an unthinned stand as a control (Stand density is 5000 trees per hectare).

Periodic annual increment of stand volume increased and then decreased with stand age, and thinning altered periodic annual increment of stand volume. The periodic annual increment of stand volume of the unthinned stand peaked at 14.6 m^3^·hm^-2^ year^-1^ at 15 years and decreased thereafter. The intensity of pre-commercial thinning was positively related to periodic annual increment of stand volume after thinning. Periodic annual increment of stand volume was 13.3%, 23.1%, and 32.4% higher at residual densities of T_E3250_, T_E2500_, and T_E1750_, respectively, compared with that of the unthinned stand (*p*<0.05). Although the periodic annual increment of stand volume of the thinned stands gradually decreased with stand age, it was always higher than that of the unthinned stand (**Figure 12A**). periodic annual increment of stand volume decreased significantly after commercial thinning but increased with stand age. Stands with higher residual density had greater periodic annual increment of stand volume. The periodic annual increment of stand volume of stands with residual densities of T_L400_, T_L278_, and T_L156_ after commercial thinning were 87.1%, 70.7%, and 46.2% higher than the productivity of the unthinned stand (*p*<0.05), respectively. However, periodic annual increment of stand volume after commercial thinning was positively correlated with residual stand density. This is in contrast to the relationship between periodic annual increment of stand volume and residual density in stands subjected to pre-commercial thinning (**Figure 12B**). Affected by the intensity of thinning, stand structural heterogeneity in pre-commercial thinning was negatively correlated with forest productivity, while stand structural heterogeneity in commercial thinning was positively correlated with forest productivity (*p*<0.05) (**Figure 13**).

## 4 Discussion

### 4.1 The effect of thinning on the quantitative maturity age

Quantitative maturity is a quantitative indicator that expresses the growth rate of a stand in absolute value and is an absolute sign of stand maturity. Determining the quantitative maturity age is a basic work for rationally organizing forestry production in terms of time and space (Liu et al., 1989). In the theory of forest management, the quantitative maturity age is considered as the cutting age of a stand, which is an important basis for determining sustainable utilization and rational harvesting (Meng, 2006). In the early stage of growth, stand volume increases with stand density. After the stand becomes closed, competition increases among the remaining living trees. When stand density and competition are in equilibrium, changes in stand volume are constrained (Binkley et al, 2010). The stand volume will decrease if the closed stand is thinned at this time. However, if the supply of habitat resources and growth space are altered to varying degrees, the individual volume of residual trees and stand volume will increase. When a new equilibrium is achieved between residual stand density and competition, the stand volume eventually reaches another plateau, and this increases the quantitative maturity age. Moreover, greater thinning intensities increase the time it takes for an equilibrium to be achieved, which increases the quantitative maturity age (Sheng, 2001). In our study, the quantitative maturity age of pre-commercial thinning stands was extended by 1 to 8 years, this is consistent with other research results on the mature period of Chinese fir plantations (Zhang et al., 2006; Huo et al., 2018). However, some studies have also found that the mature age of stand number advances with the increase of initial density, but the initial density has little effect on the average volume growth of stand quantitative mature age (He et al., 1997), which is mainly due to the fact that the fitted Chinese fir plantation has not been thinned, and the stand growth is always in a constant state without human disturbance. The quantitative maturity ages of commercially thinned stands with residual densities of 400 and 278 trees per hectare were increased by 8 and 9 years, respectively. The commercially thinned stand with a residual density of 156 trees per hectare had not yet reached the quantitative maturity age because the mean stand volume increase was much lower than the current annual increase. This is mainly due to the fact that most of the forest trees were removed by the extremely intensive commercial thinning, resulting in a small number of remaining trees in the stand, and the asymmetric competition from aboveground and underground was also weakened, and the underground root system was gradually extended, and the activity was improved. The availability of nutrient resources has been greatly improved, and nutrients are transported to the ground to grow a larger canopy, which promotes the growth of its own individual (Soares et al., 2017; Forrester, 2019). Therefore, although the average growth during the observation period did not appear the rapid growth state similar to thinning. However, the average growth has been in a continuous growth mode, and it still maintains good growth potential after 9 years of thinning.

### 4.2 Thinning and stand growth

Differences in the acquisition, absorption, and utilization of habitat resources among living trees in a stand lead to inter-individual growth variability. In particular, strong inter-tree competition in closed stands significantly limits the radial growth of individual trees and stand diameter structure also changes with stand age (Binkley et al., 2010; Sun et al., 2011). The radial growth of the few dominant trees in closed stands is not affected by adjacent trees, but that of small-sized, suppressed trees is significantly inhibited. By removing suppressed trees with limited radial growth, thinning can enhance growth and the availability of nutrients, thereby promoting the radial growth of residual trees. Moreover, the removal of small-sized trees further promotes the growth of medium- and large-diameter trees, which leads to significant changes in stand diameter structure (Zheng et al., 2020). In this study, both pre-commercial and commercial thinning accelerated increases in stand diameter. pre-commercial thinning reduced the left skewness in stand diameter structure and ultimately made the distribution in stand diameter right-skewed; the peak in the stand diameter distribution also gradually decreased. The proportions of medium- and large-diameter trees increased. These changes became more pronounced as the thinning intensity increased, which is consistent with the results of previous studies (Zhang et al., 2006; Lemay et al., 2018). Commercial thinning drastically altered stand diameter structure and resulted in an abrupt increase in the size of the initial diameter class. Stand diameter structure changed from left-skewed to right-skewed immediately after thinning. The proportion of large-sized trees in the stand was much higher than that of medium-diameter ones. Therefore, pre-commercial and commercial thinning promoted the growth of medium and large-sized Chinese fir trees, respectively.

Thinning increases the supply of habitat resources and promotes the growth of residual trees. However, the recovery of stand volume can take several years to reach its pre-thinning level when the thinning intensity is high; it might even remain consistently lower than the pre-thinning level (Arun et al., 2018). In this study, increases in the individual volume of pre-commercially and commercially thinned stands were greater than increases in the individual volume of unthinned stands. Moreover, the thinning intensity was positively related to increases in individual volume. This is consistent with the results obtained by Lu Dehao et al. in different densities of Chinese fir plantations. In this study, after thinning, the retention density was 2100 trees per hectare, and its average diameter at breast height, tree height and individual tree volume were significantly higher than those of 3100 and 4100 trees per hectare (Lu et al., 2023); at the same time, some studies have shown that the average diameter at breast height of medium-diameter timber cultivation stands is significantly larger than that of control stands when moderate pre-commercial thinning is performed once at a stand age of 12 years, and moderate thinning significantly promotes the increase of individual tree volume (Xu et al., 2014). As for the timber merchantable volume in the stand, since thinning is mainly to cut down the individual trees with poor growth in the stand, it will increase the ratio of the number of medium-diameter and large-diameter timber in the stand timber merchantable volume (Xiang, 2013; Wu et al., 2015; Song et al., 2022). In the early stage, only one pre-commercial thinning was carried out, and in the early stage of the whole stand growth cycle, although the competition intensity among individuals in the stand would increase again with the increase of the stand age, the proportion of the number of medium-diameter timber in the stand was high when the quantitative mature age was reached. However, small-diameter and some medium-diameter trees were removed in the commercial thinning stand, resulting in fewer stand trees and larger average diameters at breast height. Therefore, the proportion of large-diameter trees in the stand was the highest when the quantitative mature age was reached. Although thinning can promote individual tree volume and increase the proportion of medium and large-diameter timber, it cannot increase stand volume, which is consistent with the research results of middle-aged monoculture Chinese fir plantations (Lu et al., 2020). Thinning leads to a decrease in stand volume, especially in heavy thinning, the stand volume cannot reach the volume of unthinned stands of the same age in a short period of time (Zang et al., 2022). This is mainly related to the fact that the transition from high to low stand density reduces the intensity of intraspecific competition, and the reserved trees obtain sufficient growth space and nutrient resources. The individual tree volume and the number of trees per unit area work together on the stand volume, although reducing the stand density can promote the growth of the reserved trees, it also reduces the number of standing trees in the stand. The key to consider the stand volume is the growth of the number of reserved trees, whether the increase can supplement the accumulation of the missing number of plants (Tong et al., 2000). However, some studies have also found that when the number of individual Chinese fir trees in a stand accounts for 81.5% of the total number of trees in an unthinned stand, there is no significant difference in stand volume between the two in the retest after 3 years of thinning, which shows that light thinning is not only It can promote the increase of individual tree volume in a stand, and can also replenish the volume lost due to thinning in a short period of time (Wu et al., 2015). At the same time, when the stand volume was summed up from the existing volume and thinned volume, the volume of the thinned stand was significantly higher than that of the unthinned forest, and the volume of each stand eventually tended to the law of constant yield as the age of the stand increased (Zhang et al., 2005; Guo et al., 2019). The periodic annual increment of stand volume of pre-commercially thinned stands increased with the thinning intensity. Changes in periodic annual increment of stand volume with stand age after pre-commercial thinning were the same as those in the unthinned stand. By contrast, periodic annual increment of stand volume rapidly increased after commercial thinning and decreased with the thinning intensity. Changes in the periodic annual increment of stand volume of the unthinned stand were opposite those observed in the periodic annual increment of stand volume of thinned stands. This also explains differences in stand volumes under pre-commercial and commercial thinning.

### 4.3 Stand structure and forest productivity

The regulation of stand density reduces the stand canopy density to a certain extent, releases the growth and nutrient space, increases the radiation flux of the forest canopy, promotes the decomposition and circulation of litter, thereby promoting the growth of trees, making the stand The internal diameter step rapidly shifted from small to large, and then had a significant impact on the stand structure (Lin et al., 2016). Competition between living trees in unthinned Chinese fir stands gradually increased with stand age, and dominant trees utilized more resources because of their dominant position. By contrast, the growth potential of suppressed trees gradually decreased because of their inability to obtain sufficient resources to maintain normal growth. This increases the size differentiation in the stand and thus stand structural heterogeneity (Yang et al., 2019). After closed stands were thinned by removing poorly growing trees, the difference in size between the residual trees was significantly reduced. Moreover, the thinning intensity was inversely related to the size difference between residual trees and stand structural heterogeneity (Lemire et al., 2020; Moreau et al., 2020). However, competition between residual trees and structural heterogeneity increase with stand age after thinning. This is basically consistent with post-thinning changes observed in the structure of white oak (*Quercus alba* L.) and eucalyptus (*E. grandis* × *E. urophylla*) stands (Soares et al., 2017; Wang et al., 2021). Although post-thinning differentiation occurs again with the growth of residual trees, the magnitude of differentiation is much lower after thinning than without thinning. The main reason is that habitat resource-related growth restrictions are reduced, which decreases competition between residual trees and results in less differentiation. The thinning intensity is inversely related to the size difference between residual trees and stand structural heterogeneity.

Stand structure is mainly related to the degree of differentiation among trees in the stand. When there is considerable differentiation between trees in a high-density stand, stand structural heterogeneity is increased, and forest productivity is lower (Duduman, 2011). Thinning is mainly performed to remove suppressed trees in the stand. This reduces the size difference between residual trees, as well as the intensity of competition for growth resources, which increases forest productivity. In pre-commercially thinned Chinese fir stands, the thinning intensity is inversely related to stand structural heterogeneity and positively related to productivity (Soares et al., 2016; Mhamdi et al., 2021). Stand structural heterogeneity and productivity were much lower under commercial thinning than under pre-commercial thinning. However, stand structural heterogeneity was positively correlated with forest productivity after commercial thinning. This is because limitations to the growth of residual dominant trees associated with reductions in the availability of habitat resources were basically eliminated by commercial thinning. In other words, residual stand density was positively related to forest productivity.

## 5 Conclusions

Timely and moderate thinning is an important measure for improving timber quality and stand yield. The results of this study show that thinning at different times and intensities affected the quantitative maturity age, diameter distribution, structure, and growth of Chinese fir stands. Specifically, thinning reduced stand structural heterogeneity, promoted increases in stand diameter, and increased the medium-sized and large-sized timber merchantable volumes. Individual volume, forest productivity, and the quantitative maturity age increased with thinning intensity. In the Chinese fir plantations in the hilly terrain of the northern Chinese fir production area, when pre-commercial thinning was performed nine years after the plantation was established at a residual density of 1,750 trees per hectare, the quantitative maturity age was 30 years, medium-sized timber accounted for 75.2% of all trees, and the stand volume was 667.9 m^3^ per hectare. This thinning strategy is favorable for producing medium-sized Chinese fir timber. A residual density of 400 trees per hectare is advisable for commercial thinning. The quantitative maturity age was 31 years, large-sized timber accounted for 76.6% of all trees, and the stand volume was 574.5 m^3^ per hectare. This thinning strategy is favorable for producing large-sized Chinese fir timber.

## Data availability statement

The original contributions presented in the study are included in the article/supplementary material, further inquiries can be directed to the corresponding author.

## Author contributions

KS analyzed the data and drafted the manuscript. HS and LF designed the study and supervised the work. GL, ZW, and ZT contributed to the installation of the sampling plots and prepared the data. All authors revised drafts and approved the final manuscript. All authors contributed to the article.
